# Vasectomy in Spermatorrhea

**Published:** 1902-02

**Authors:** 


### Vasectomy in Spermatorrhea.

In the *New Orleans Medical and Surgical Journal,* with reference to the effect of vasectomy upon spermatorrhea, Dr. G. Frank Lydston says: "In certain cases of intractable spermatorrhea vasectomy is likely to produce excellent results. It benefits in three ways: (1) By preventing a serious drain upon the system incidental to the seminal loss *per se;* (2) by the moral effect of the cessation of the escape of semen from the patient; (3) it benefits the chronic prostatic and seminal vesiculitis with which the spermatorrhea is likely to be associated." He does not see any real objection to the operation of vasectomy in cases of intractable spermatorrhea. The individual is already impotent and his general health is sure to be much improved by the cessation of the seminal loss and the correction of the existing prostatic disease with which the spermatorrhea is usually associated.

